# COVID-19 diagnostic testing and vaccinations among First Nations in Manitoba: A nations-based retrospective cohort study using linked administrative data, 2020–2021

**DOI:** 10.1371/journal.pmed.1004348

**Published:** 2024-02-16

**Authors:** Nathan C. Nickel, Wanda Phillips-Beck, Jennifer E. Enns, Okechukwu Ekuma, Carole Taylor, Sarah Fileatreault, Nkiru Eze, Leona Star, Josée Lavoie, Alan Katz, Marni Brownell, Alyson Mahar, Marcelo Urquia, Dan Chateau, Lisa Lix, Mariette Chartier, Emily Brownell, Miyosha Tso Deh, Anita Durksen, Razvan Romanescu

**Affiliations:** 1 Manitoba Centre for Health Policy, Dept of Community Health Sciences, Rady Faculty of Health Sciences, University of Manitoba, Winnipeg, Canada; 2 First Nations Health and Social Secretariat of Manitoba, Winnipeg, Canada; 3 Dept of Community Health Sciences, Rady Faculty of Health Sciences, University of Manitoba, Winnipeg, Canada; 4 Dept of Family Medicine, Rady Faculty of Health Sciences, University of Manitoba, Winnipeg, Canada; 5 School of Nursing, Faculty of Health Sciences, Queen’s University, Kingston, Canada; 6 National Centre for Epidemiology and Population Health, College of Health and Medicine, Australia National University, Canberra, Australia; 7 George and Fay Yee Centre for Healthcare Innovation, University of Manitoba, Winnipeg, Canada; Harvard University, UNITED STATES

## Abstract

**Background:**

Differential access to healthcare has contributed to a higher burden of illness and mortality among First Nations compared to other people in Canada. Throughout the Coronavirus Disease 2019 (COVID-19) pandemic, First Nations organizations in Manitoba partnered with public health and Manitoba government officials to ensure First Nations had early, equitable and culturally safe access to COVID-19 diagnostic testing and vaccination. In this study, we examined whether prioritizing First Nations for vaccination was associated with faster uptake of COVID-19 vaccines among First Nations versus All Other Manitobans (AOM).

**Methods and findings:**

In this retrospective cohort study, we used linked, whole-population administrative data from the Manitoba healthcare system (February 2020 to December 2021) to determine rates of COVID-19 diagnostic testing, infection, and vaccination, and used adjusted restricted mean survival time (RMST) models to test whether First Nations received their first and second vaccine doses more quickly than other Manitobans.

The cohort comprised 114,816 First Nations (50.6% female) and 1,262,760 AOM (50.1% female). First Nations were younger (72.3% were age 0 to 39 years) compared to AOM (51% were age 0 to 39 years) and were overrepresented in the lowest 2 income quintiles (81.6% versus 35.6% for AOM). The 2 groups had a similar burden of comorbidities (65.8% of First Nations had none and 6.3% had 3 or more; 65.9% of AOM had none and 6.0% had 3 or more) and existing mental disorders (36.9% of First Nations were diagnosed with a mood/anxiety disorder, psychosis, personality disorder, or substance use disorder versus 35.2% of AOM).

First Nations had crude infection rates of up to 17.20 (95% CI 17.15 to 17.24) COVID-19 infections/1,000 person-months compared with up to 6.24 (95% CI 6.16 to 6.32) infections/1,000 person-months among AOM. First Nations had crude diagnostic testing rates of up to 103.19 (95% CI 103.06 to 103.32) diagnostic COVID-19 tests/1,000 person-months compared with up to 61.52 (95% CI 61.47 to 61.57) tests/1,000 person-months among AOM. Prioritizing First Nations to receive vaccines was associated with faster vaccine uptake among First Nations versus other Manitobans. After adjusting for age, sex, income, region of residence, mental health conditions, and comorbidities, we found that First Nations residents received their first vaccine dose an average of 15.5 (95% CI 14.9 to 16.0) days sooner and their second dose 13.9 (95% CI 13.3 to 14.5) days sooner than other Manitobans in the same age group.

The study was limited by the discontinuation of population-based COVID-19 testing and data collection in December 2021. As well, it would have been valuable to have contextual data on potential barriers to COVID-19 testing or vaccination, including, for example, information on social and structural barriers faced by Indigenous and other racialized people, or the distrust Indigenous people may have in governments due to historical harms.

**Conclusion:**

In this study, we observed that the partnered COVID-19 response between First Nations and the Manitoba government, which oversaw creation and enactment of policies prioritizing First Nations for vaccines, was associated with vaccine acceptance and quick uptake among First Nations. This approach may serve as a useful framework for future public health efforts in Manitoba and other jurisdictions across Canada.

## Abstract 2

Nathan C Nickel, Wanda Phillips-Beck and colleagues assess whether First Nations-led COVID-19 protective measures were associated with improved access to COVID-19 testing and vaccination compared to all other Manitobans.

## Author summary

**Why was this study done?:**

- Historically, pandemics have disproportionately affected marginalized groups, such as Indigenous Peoples, in a negative way.
- Evidence of the disporportionate burden of Coronavirus Disease 2019 (COVID-19) on First Nations people in the province of Manitoba, Canada, became clear when collection of Indigenous identifiers began in May 2020.
- First Nations organizations in Manitoba collaborated with the provincial government to develop and implement COVID-19 testing and vaccine administration strategies to improve access for First Nations people living in Manitoba.
- We wanted to know if First Nations-led efforts to improve access to COVID-19 protective measures were associated with higher COVID-19 testing rates, lower COVID-19 infection rates, and higher vaccination rates for First Nations people compared to other Manitobans.

**What did the researchers do and find?:**

- We used a “Nations-based approach,” meaning different Indigenous populations were not compared to one another in recognition of each population’s unique characteristics. Instead, First Nations were compared to All Other Manitobans (AOM).
- We used whole-population data from the province of Manitoba to compare diagnostic testing, infection, and vaccination rates among Manitoba First Nations and AOM and examined how quickly vaccines were administered to each group once people were eligible to receive them.
- From this work, we found that while being prioritized, First Nations had higher testing rates, infection rates, and vaccination rates compared to AOM. As well, First Nations were vaccinated more quickly than AOM after becoming eligible for both first and second doses of the COVID-19 vaccine.

**What do these finding mean?:**

- Our results suggest that First Nations-led advocacy and efforts to prioritize First Nations people for COVID-19 protective measures were associated with faster vaccine access and uptake by First Nations people and this may have prevented many severe illnesses and deaths.
- Availability of Indigenous identifiers through collaborative data sovereignty agreements has made this work possible, providing a clearer picture of the outcomes of First Nations’ self-determination and public health sovereignty approaches.
- However, we did not have data on potential social and structural barriers to COVID-19 testing and vaccination faced by Indigenous and racialized people and this is an important area of future research.
- Broadly speaking, informative, inclusive, and participatory decision-making and policy creation are key to protecting health and preventing the spread of communicable illnesses among Indigenous populations.

## Introduction

Globally, Indigenous peoples are often disproportionately affected by epidemics, pandemics, and other health crises [1,2]. Contributing factors that increase the potential for severe outcomes can include inequitable access to clean water, quality housing infrastructure, and medical services; moreover, Indigenous peoples may also experience discrimination in healthcare settings, thus compromising the standard of care they receive and discouraging them from accessing services when they are available. The negative impacts of under-funding of Indigenous health and social services also become more evident in public health crises like the Coronavirus Disease 2019 (COVID-19) pandemic and have been the subject of numerous policy papers recommending immediate action to protect and support Indigenous communities in navigating the ongoing challenges and recovery efforts related to the pandemic [1,2].

In Canada, the COVID-19 virus posed a greater health risk to Indigenous peoples (First Nations, Métis, and Inuit) than to non-Indigenous Canadians since it was first detected in early 2020. Throughout the early pandemic stages, First Nations living in rural and urban communities had a higher risk of contracting COVID-19 and were more likely than the general population of Canada to experience severe outcomes following infection [3]. Many feared that this public health crisis would be a repeat of the H1N1 pandemic in 2009 [4], where the actual and extraordinarily high burden faced by First Nations peoples in Manitoba was initally unknown because First Nations-specific data were lacking. But through public health advocacy and support from First Nations leaders, collection of Indigenous identifiers in Manitoba became mandatory in May 2020 [5], and the disproportionate burden of COVID-19 on First Nations peoples became clear: in 2020, First Nations people accounted for 17% of deaths, 33% of hospitalizations, and 55% of intensive care unit (ICU) admissions from COVID-19 [6], while representing less than 12% of the population in Manitoba [7]. During another wave of COVID-19 in May 2021, approximately 40% of deaths were Indigenous peoples in Manitoba [8].

Historically, pandemics have disproportionately affected marginalized groups in a negative way [9–12]. During the H1N1 outbreak in 2009, First Nations experienced higher rates of hospitalizations and ICU admissions compared to other people in Canada [11,12], attributed at least in part to systemic and structural factors contributing to poverty, crowded living spaces, and lower quality of health services [13,14]. The H1N1 pandemic also highlighted how excluding Indigenous leaders from public health planning and limiting their access to population health data exacerbated existing health inequities between First Nations and others in Canada and prevented timely equity-informed policy adjustments [15,16]. Building from this previous experience during the COVID-19 pandemic, public health officials and First Nations leadership in Manitoba worked together to optimize protective measures and minimize health inequities for First Nations.

Despite Indigenous Peoples’ right to self-government in Canada being recognized and affirmed in the Constitution Act [17], First Nations continue to be impacted by systemic and structural barriers to high-quality healthcare [18]. Government’s failure to meet its treaty obligations [19] has prompted First Nations organizations in Manitoba to assume responsibility for improving quality of healthcare services [20] and providing leadership in health policy development and implementation [21]. When the COVID-19 pandemic began in Canada, Manitoba First Nations leaders recognized the risk to their people and leveraged their existing infrastructure to form the Manitoba First Nations Pandemic Response Co-ordination Team, a partnership between the Assembly of Manitoba Chiefs, Manitoba Keewatinowi Okimakinak, the First Nations Health and Social Secretariat of Manitoba (FNHSSM), and Keewatinohk Inniniw Minoayawin, and coordinated by Ongomiizwin Health Services at the University of Manitoba [22]. Throughout the COVID-19 pandemic, the First Nations Pandemic Response Co-ordination Team has been pivotal in public health decision-making, providing reliable and culturally appropriate information, coordinating First Nations-led services, and advocating for priority testing and vaccination for First Nations [21–24]. As part of their efforts, multidisciplinary Rapid Response Teams were deployed to First Nations to conduct diagnostic testing, contact tracing, and vaccinations. First Nations leadership also collaborated with the Manitoba government to develop and implement COVID-19 testing and vaccine administration strategies to improve access for those that faced additional barriers (e.g., mobility and transportation challenges, reaching unhoused people) [25,26]. And when the vaccine first became available in late 2020, First Nations were among the Manitobans soon considered to be at higher risk of infection (along with healthcare workers, older adults living in residential care facilities, and immunocompromised people), and thus were offered priority access to vaccination [25,27].

Against this backdrop of First Nations-led efforts to improve access to COVID-19 protective measures, we tested the hypothesis that First Nations in Manitoba would have higher COVID-19 testing rates, lower COVID-19 infection rates, and higher vaccination rates than other Manitobans, or at least equivalent rates. Moreover, because they had priority access to vaccination, we hypothesized that First Nations would have faster uptake of vaccines than other Manitobans once they became eligible to receive them.

## Methods

In this retrospective cohort study, we used linked whole-population administrative data from the province of Manitoba, Canada to determine whether First Nations-led COVID-19 protective measures were associated with improved access to COVID-19 vaccination. We compared diagnostic testing, infection, and vaccination rates among Manitoba First Nations and all other Manitobans (AOM), and examined how quickly vaccines were administered to each group once people were eligible to receive them. This study is reported as per the REporting of studies Conducted using Observational Routinely collected Data (RECORD) guidelines [28]. A published prospective protocol provides details on our planned analyses [29].

### Nations-based study approach

Indigenous scholars and policymakers across Canada have argued that no single entity can represent or serve all Indigenous Peoples, and that specific First Nations, Métis and Inuit responses are required for different policy areas and contexts [30]. In this study, we aim to decolonize COVID-19 research, which in the broadest sense involves sharing power: the power to know, based on being in the world, and the power to do, according to one’s learned and sensory interactions with physical environments [31]. Decolonizing research in this study meant adopting a Nations-based framework [11] in which no comparisons were made between First Nations, Métis, and Inuit people; instead, First Nations outcomes were compared to AOM outcomes. Our decolonizing approach also included active involvement of First Nations researchers in the project as co-investigators and decision-makers, from the proposal stage to analysis and dissemination. This approach adheres to Indigenous data sovereignty principles [30,32] and OCAP principles [33], and has been adopted at the direction of our First Nations research partners. It is also endorsed by Indigenous research organizations and advocates, as it acknowledges the distinct histories, interests, and priorities of First Nations as well as distinct policy efforts and implications of the unique relationship of First Nations with the Crown [11–14].

### Data sources

We used routinely collected administrative health data from the Manitoba Population Research Data Repository at the Manitoba Centre for Health Policy, University of Manitoba. The Repository contains linkable, de-identified, and individual-level administrative records on contacts with the health system, social services, the education system, and the justice system for virtually the entire population of Manitoba (>99.9% of the Manitoba population). The records are de-identified (names and addresses removed), but are linkable to a central population registry by way of a scrambled numeric identifier (each person’s 9-digit personal health identification number) attached to each record by a third party before the records are transferred to the Repository. Through the whole-population registry, records from various databases can then be linked across domains and over time to conduct cross-sectional and longitudinal studies. The databases are subject to extensive quality assessment to ensure that standards for linkage accuracy, completeness, and other data quality metrics are met before they are used for population health research [34].

The specific databases accessed for this study were the Manitoba Health Insurance Registry (demographic characteristics of the Manitoba population); Canada Census and Statistics Canada public use data (small area-level income and postal codes); physician visit claims, hospital abstracts, and prescriptions from community pharmacies (primary healthcare, hospitalizations, and prescriptions, and these were also used to calculate the Charlson comorbidity index); and laboratory data from Diagnostic Services Manitoba (COVID-19 PCR tests, test results, and vaccinations). Information on First Nations status came from the Manitoba First Nations Research File, a registry containing information on Manitoba First Nations individuals registered under the Indian Act as “status Indians.” More details are available in **S1 Table**. The registry was created as part of a tripartite agreement between FNHSSM, the Manitoba Centre for Health Policy, and Manitoba Health, and draws information from the Status Verification System at the First Nations and Inuit Health Branch, Indigenous Services Canada. The Manitoba First Nations Research File was cross-referenced with 4 other databases in the Repository that include self-reported information on First Nations identity (the Universal Newborn Screen, Healthy Baby Program Data, Employment and Income Assistance, and the Early Development Instrument databases). All individuals identified as First Nations or as a mother of a First Nations person (using existing mother–child linkages in the Repository) in at least one of these 5 databases were included in the cohort.

### Study cohort

**Fig 1:**
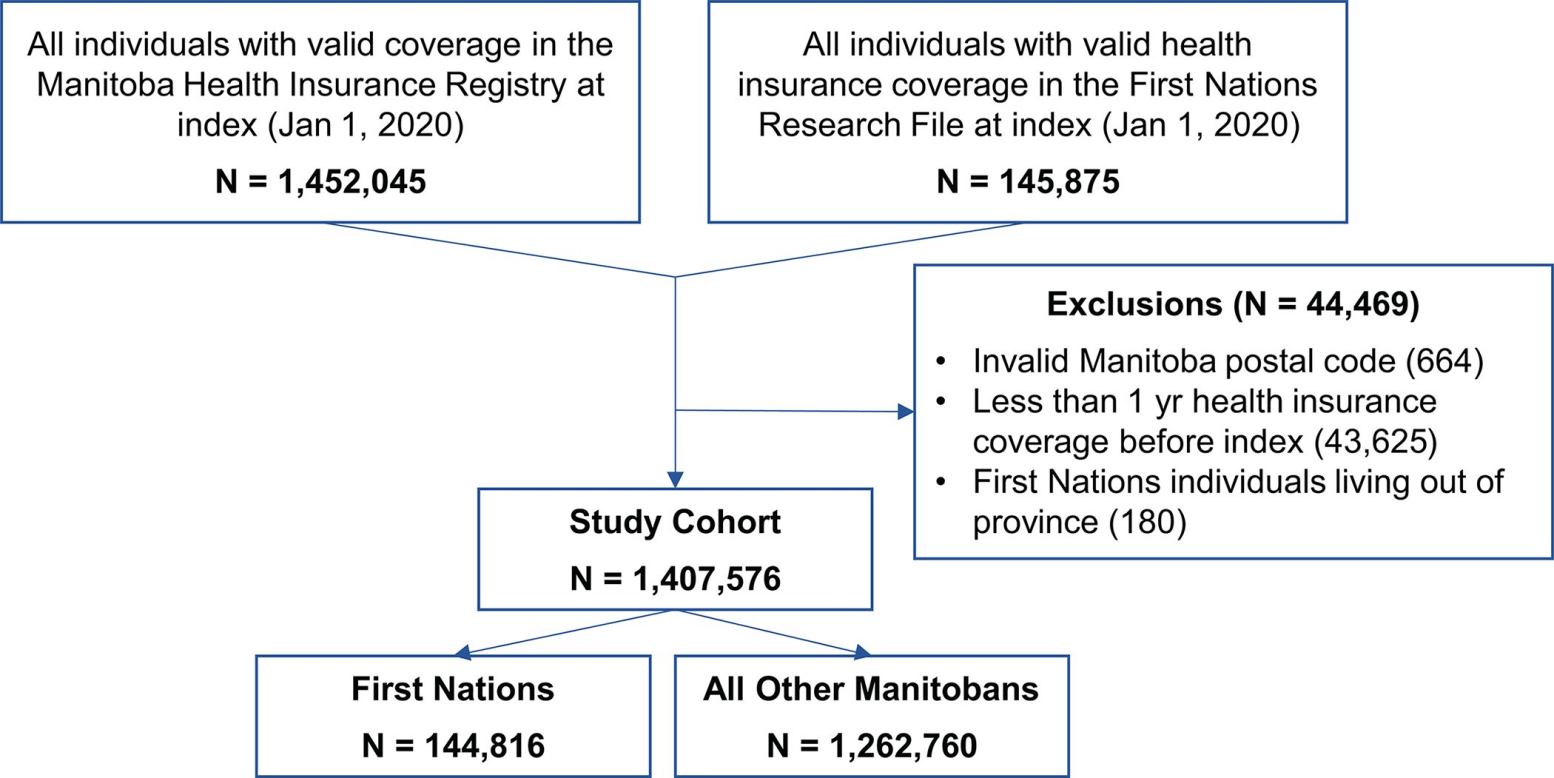
Study cohort development.

Cohort development is shown in **Fig 1**. We started with all Manitoba residents (First Nations and other Manitobans) who had at least 1 year of valid provincial health insurance coverage prior to the index date (January 1, 2020) and who were living in Manitoba at any point from January 1, 2020 to December 31, 2021. We excluded individuals for whom we did not have a valid postal code, who had less than a year of coverage before the index date, or who were living out of province during the study period, then stratified First Nations and AOM into separate study groups. The final cohort comprised 144,816 First Nations and 1,262,760 other Manitobans.

### Variables

We obtained information on age, sex, region of residence (urban/rural) from the Manitoba Health Insurance Registry, neighborhood-level average income from the Canada Census, and postal code of residence from Statistics Canada to describe the cohort and adjust for baseline differences between study groups. Data from physician visit claims, hospitalizations, and prescription medications were used to identify people who sought provincially funded mental health-related services and to quantify physical health comorbidities using the Charlson comorbidity index; these were included as covariates in our statistical models.

The primary outcomes for the study were the following: (1) rates of COVID-19 diagnostic testing, infection, and vaccination; and (2) time to vaccination after becoming eligible for the COVID-19 vaccine. A diagnostic test was defined as a PCR-based COVID-19 test administered at a provincial testing site and processed at a provincial laboratory; during the study period, the vast majority of COVID-19 testing was done at provincial sites since home-administered rapid antigen testing was not yet widely available in Manitoba. COVID-19 infections (positive/negative) were determined from the laboratory test results. Vaccination records in Manitoba include information on the type/brand of vaccine administered, the date of vaccination, and the number of doses received. We calculated crude monthly testing and infection rates using laboratory data from February 2020 to December 2021 and vaccination rates using vaccination data from December 2020 to December 2021. These timeframes align with the start of the pandemic in Manitoba (February/March 2020), the earliest date vaccines were available (December 2020), and the end of population-based diagnostic PCR testing for COVID-19 (December 2021). The denominator for the testing and infection rates was the entire population of First Nations or AOM, respectively, who met the inclusion criteria for the study cohort. For vaccination, the denominator was the age-eligible population in each study group (vaccination was rolled out from highest to lowest age group in both First Nations and AOM over the course of 2021).

### Statistical analyses

All analyses were done in SAS/STAT software version 15.1 [35]. We generated descriptive statistics for the cohort using counts and proportions. To adjust for differences in sociodemographic characteristics when comparing First Nations with AOM, we used standardized differences with an a priori cut off of 10% to signal significant differences between groups. We used a negative binomial distribution with log link function to examine the association between being First Nations or AOM and the rate of COVID-19 diagnostic testing, infection, and vaccination. The log of person-days as an offset was used to account for partial residency. Model goodness of fit was assessed by dividing the deviance and the scaled deviance by their degrees of freedom and values were within the acceptable range. We also considered zero-inflated models, but the negative binomial distribution was a better fit. The covariates included in the models were: First Nations or AOM, age, sex, region of residence (urban/rural), income quintile, any mental health condition, and the Charlson comorbidity index.

We also examined the association between being First Nations or AOM and the time (in days) to first and second vaccination. We first used Cox proportional hazard models to assess these associations, but the assumptions of proportional hazards were violated in our study, prompting us to seek another approach. We selected RMST models, which are a good alternative to Cox proportional hazards models if the assumptions of proportionality of hazards are violated [36,37]. RMST models generate the average time from an event up until a defined time point (i.e., the numerical expression of the area under the Kaplan–Meier survival curve) [36,37]. We restricted the mean survival time calculation to 396 days from December 1, 2020, for both first and second vaccinations. The covariates included in these models are the same as those included in the negative binomial distribution above. With the RMST models, we tested whether First Nations were vaccinated sooner than AOM while First Nations had priority access to vaccination. We also determined whether these public health efforts to improve First Nations’ access to vaccines had an effect across different age groups, since prioritization in Manitoba was based on age.

### Ethics

The study was approved by the Health Information Research Governance Committee of the First Nations Health and Social Secretariat of Manitoba (2020), the University of Manitoba Human Research Ethics Board (HS24133-H2020:345), and the Health Information Privacy Committee of the Manitoba Government (HIPC No. 2020/2021-25).

## Results

### Cohort characteristics

The study cohort sociodemographic characteristics are presented in **Table 1**. In addition to basic demographic variables, we also considered a number of factors that could potentially influence people’s ease of access to COVID-19 testing or vaccination, such as comorbidity and mental health. The sex distribution in the cohort was similar between First Nations and AOM. First Nations tended to be younger than AOM (72.3% of First Nations were younger than 40 years versus 51.1% of AOM). First Nations were overrepresented (81.6% of individuals) in the lowest 2 income quintiles, whereas AOM were more evenly distributed across the quintiles. The majority of First Nations individuals (77.8%) lived in rural areas while only 35.3% of AOM were rural residents. The 2 groups had a similar burden of comorbidities, although a slightly higher proportion of First Nations (6.3%) had a Charlson comorbidity index of 3 or higher compared to AOM (6.0%). The prevalence of mental disorders was also similar between groups, although First Nations had a higher prevalence of substance use disorder than AOM (9.9% versus 2.8%).

**Table 1:** Sociodemographic characteristics for Manitoba First Nations and All Other Manitobans. Counts, proportions, and standardized differences between groups.

	Manitoba First Nations *N* = 114,816		All Other Manitobans *N* = 1,262,760		
	*n*	%	*n*	%	Std difference
**Sex**					
Female	73,226	50.6	633,119	50.1	0.01
Male	71,589	49.4	629,612	49.9	0.01
**Age group**					
0–19	59,778	41.3	297,126	23.5	0.39
20–39	44,908	31.0	347,491	27.5	0.08
40–59	28,544	19.7	321,888	25.5	0.14
60–79	10,672	7.4	240,469	19.0	0.35
80+	914	0.6	55,786	4.4	0.24
**Income quintile**					
Q1 (Lowest)	72,757	50.2	206,615	16.4	0.77
Q2	45,529	31.4	241,835	19.2	0.29
Q3	12,890	8.9	264,797	21.0	0.34
Q4	7,142	4.9	268,908	21.3	0.50
Q5 (Highest)	5,388	3.7	269,897	21.4	0.55
Not found	1,110	0.8	10,708	0.9	0.01
**Geography**					
Urban	32,101	22.2	816,744	64.7	0.95
Rural	112,715	77.8	446,016	35.3	0.95
**Charlson comorbidity index** (in the last 5 years)					
0	95,316	65.8	832,236	65.9	0.00
1	32,270	22.3	265,647	21.0	0.03
2	8,109	5.6	89,481	7.1	0.06
3+	9,121	6.3	75,396	6.0	0.01
**Mental disorder diagnosis** (in the last 5 years)					
Any mental disorder	53,432	36.9	443,940	35.2	0.04
Mood/anxiety disorder	25,841	17.8	235,338	18.6	0.02
Psychosis	3,112	2.2	15,899	1.3	0.07
Personality disorder	1,410	1.0	8,400	0.7	0.03
Substance use disorder	14,319	9.9	35,869	2.8	0.29

### Crude monthly rates of COVID-19 diagnostic testing, infection, and vaccination

**Fig 2:**
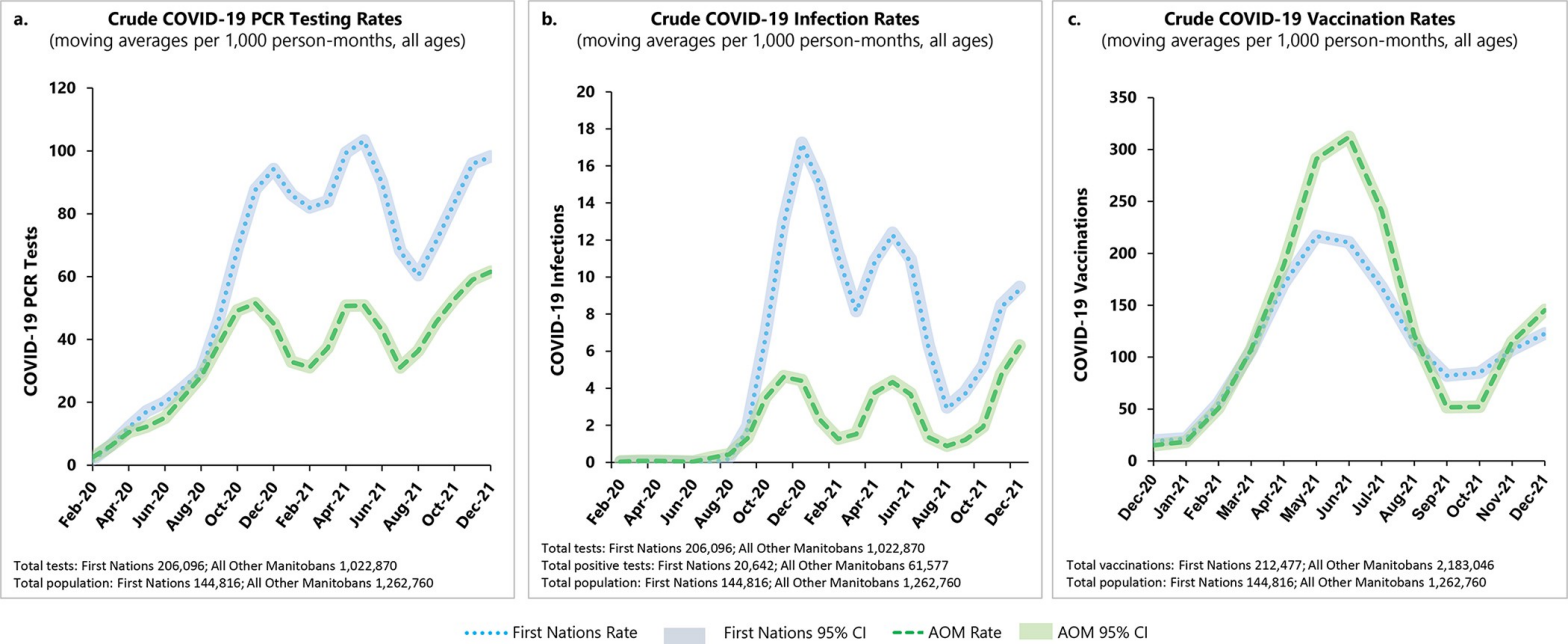
Crude monthly moving averages of COVID-19 infection, testing, and vaccination rates among Manitoba First Nations and AOM. February 2020 to December 2021. Rates per 1,000 person-months with 95% confidence intervals. Note differences in axis scales in (a–c). AOM, All Other Manitobans; COVID-19, Coronavirus Disease 2019.

As seen in **Fig 2**, rates of diagnostic testing and confirmed infections rose quickly in August to October 2020 during the first major wave of COVID-19 in Manitoba, and again in February to April 2021 during the second major wave. First Nations had crude diagnostic testing rates of up to 103.19 (95% CI 103.06 to 103.32) diagnostic COVID-19 tests/1,000 person-months compared with a maximum rate of 61.52 (95% CI 61.47 to 61.57) tests/1,000 person-months among AOM (**Fig 2A**). First Nations had crude infection rates of up to 17.20 (95% CI 17.15 to 17.24) COVID-19 infections/1,000 person-months compared with a maximum rate of 6.24 (95% CI 6.16 to 6.32) infections/1,000 person-months among AOM (**Fig 2B**). Throughout the pandemic, First Nations testing rates were typically 2 to 3 times higher than among AOM, and infection rates among First Nations were as much as 4 times higher than AOM during the first wave and up to 3 times higher during the second wave. Test positivity rates among First Nations were also consistently elevated compared to AOM during the major waves of COVID-19 in Manitoba (**S1 Fig**).

Vaccination first became available in December 2020 for select groups of Manitobans at highest risk of poor COVID-19 outcomes (including First Nations), and then became more widely available in April 2021 for the general population, starting with older and then younger groups of Manitobans. Individuals aged 12 and younger were not eligible for vaccination until after the study ended. The vaccination rate rose steeply for both First Nations and AOM from January to April 2021 (**Fig 2c**), then the First Nations rate peaked at 216.62 (95% CI 216.44 to 216.80) vaccinations/1,000 person-months. The AOM vaccination rate continued to climb until May to June 2021, reaching a peak of 312.12 (95% CI 311.97 to 312.27) vaccinations/1,000 person-months. Over June to August 2021, both groups’ vaccination rates declined as most Manitobans had by then received at least 1 dose and vaccination outreach efforts slowed. **S2 Table** presents more details on the individual data points in **Fig 2**.

### Adjusted COVID-19 vaccination rates

**Fig 3:**
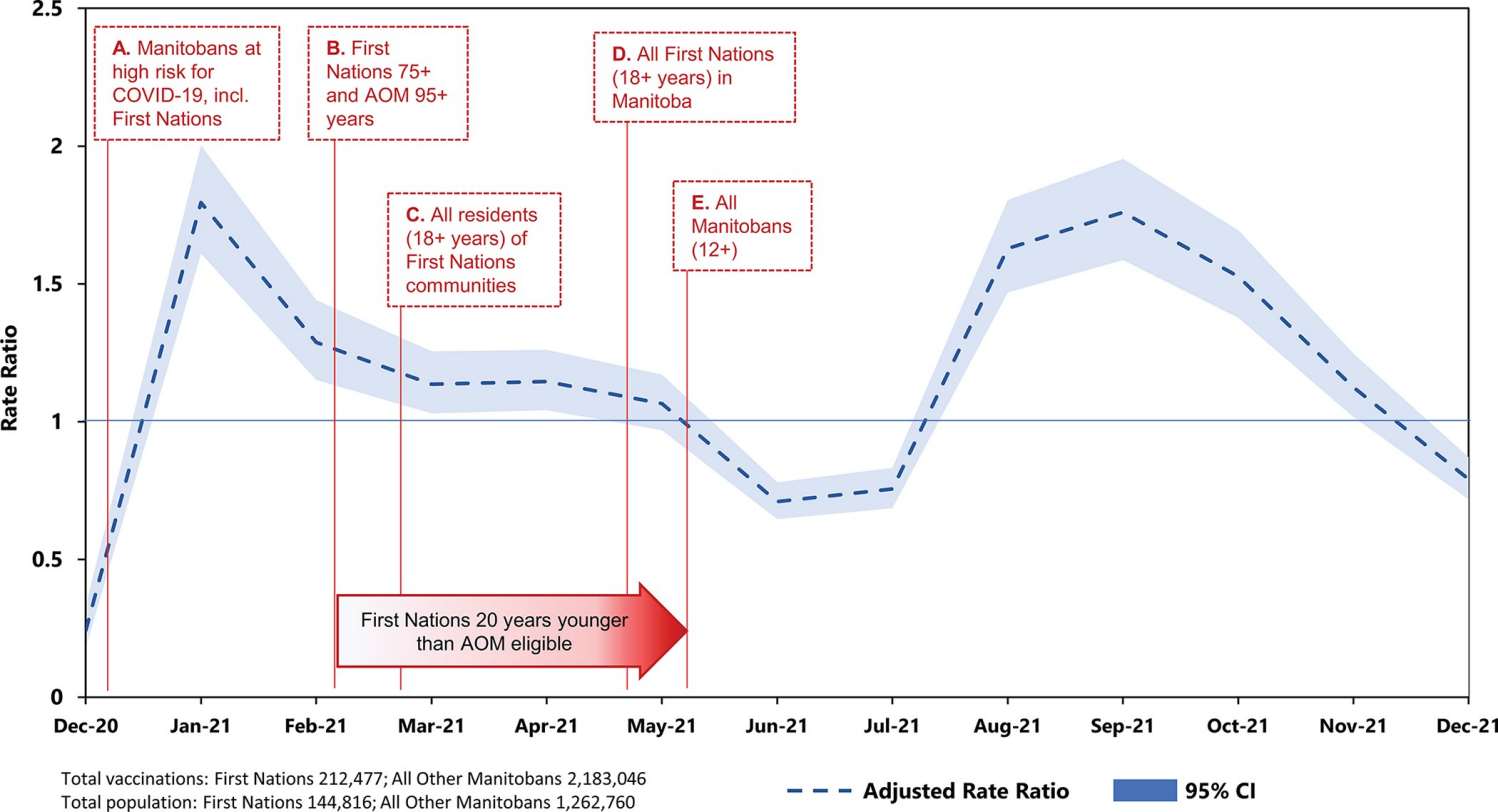
Adjusted COVID-19 vaccination rate ratios for First Nations and AOM with 95% CIs. December 2020–December 2021. Red labels denote when specific groups of Manitobans became eligible for vaccination. (A) Select Manitobans at highest risk of COVID-19 (e.g., healthcare workers, immunocompromised individuals). (B) First Nations 75+ and AOM 95+ years. (C) All residents (18+ years) of First Nations communities. (D) All First Nations (18+ years) living in Manitoba. (E) All Manitobans (12+). Rates are adjusted for age, sex, income, region of residence, mental health conditions, and Charlson comorbidity index. AOM, All Other Manitobans; COVID-19, Coronavirus Disease 2019.

After adjusting for age, sex, income, region of residence, mental health conditions, and Charlson comorbidity index, we calculated rate ratios for vaccinations received by First Nations versus AOM (**Fig 3**). The letters A–E in **Fig 3** denote key dates for vaccine eligibility. The public health policy was designed so that First Nations were eligible for vaccination at the same time as AOM who were 20 years older, thus ensuring that First Nations had priority access to vaccination at each age-based stage of the vaccine rollout. First Nations living in First Nations communities were also prioritized until all first doses were administered.

Starting at A, vaccines were available to Manitobans at high risk of exposure and/or severe outcomes of COVID-19, including healthcare workers, older adults in residential care facilities, immunocompromised people, and First Nations age 75+ years living in First Nations communities. At B, all First Nations age 75+ years and AOM age 95+ were eligible for vaccination. The rollout by age group for First Nations continued until early March (C) and early May (D), at which point all First Nations adults were eligible for vaccination. For nearly all of this period (A–D), there were higher relative rates of vaccination among First Nations than AOM, peaking at an adjusted rate ratio of 1.80 (95% CI 1.61 to 2.00) vaccinations/1,000 person-months. From D and E, when the majority of First Nations had received at least their first dose of vaccine and the youngest age groups of AOM became eligible, AOM had higher relative rates, with an adjusted rate ratio of 0.71 (95% CI 0.65 to 0.78) vaccinations/1,000 person-months. The second large peak in August to September 2021 onwards (adjusted rate ratio 1.76 [95% CI 1.59 to 1.95] vaccinations/1,000 person-months) likely reflects second or third doses of the vaccine for many First Nations. Crude and adjusted rate ratios and 95% confidence intervals for all individual data points are provided in **S3 Table**.

### Access to COVID-19 vaccination

**Fig 4:**
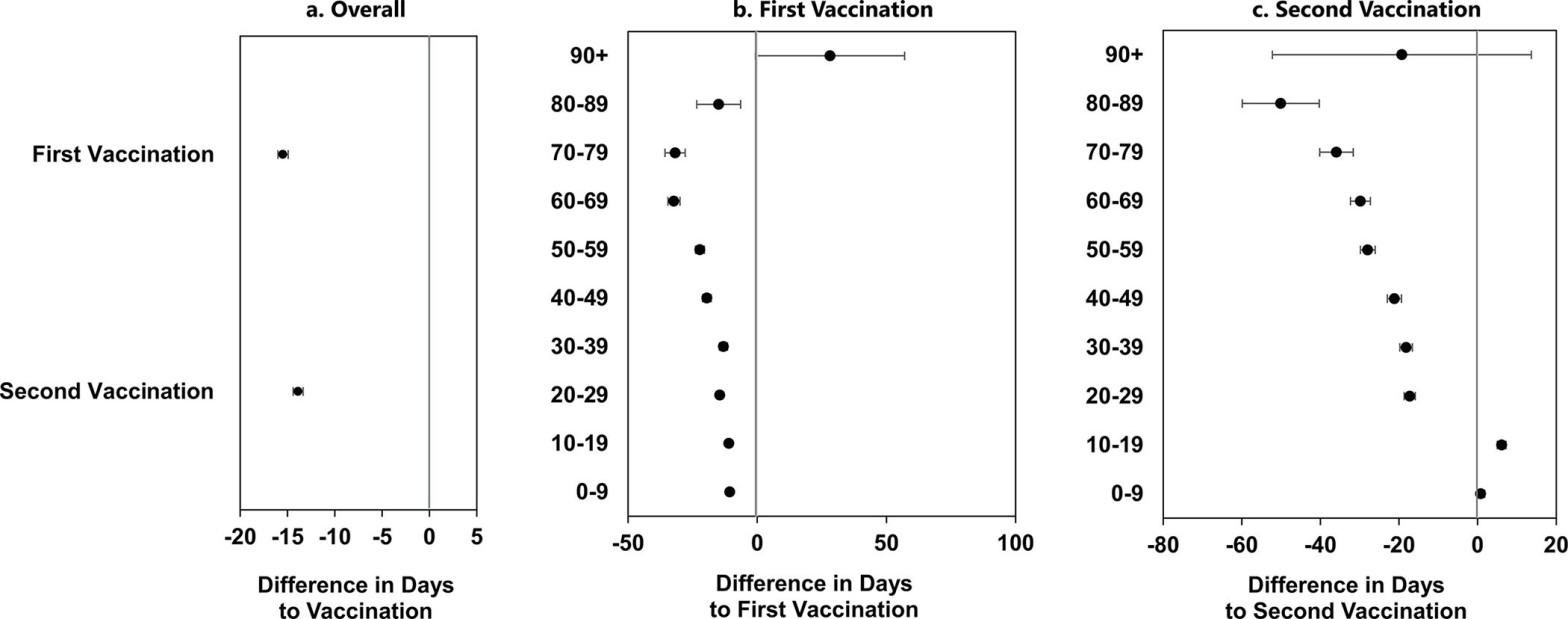
Time to vaccination for First Nations and AOM, overall and by age. December 2020–December 2021. RMST estimate with 95% CIs. Tau (total time period) = 396 days. Data values for this figure are presented in S3 Table. AOM, All Other Manitobans; RMST, restricted mean survival time.

In **Fig 4**, we present the mean difference in days between First Nations and AOM in receipt of first and second vaccinations. **Fig 4A** shows that First Nations received their first dose of vaccine an average of 15.47 (95% CI 14.94 to 16.00) days sooner than AOM, and their second dose an average of 13.88 (95% CI 13.26 to 14.50) days sooner. Because public health policy in Manitoba prioritized individuals based on age group, we also examined time until first and second vaccine doses for each 10-year age group; this is shown in **Fig 4B** and **4C**, respectively. Nearly all First Nations age groups received both vaccine doses substantially sooner than AOM, demonstrating that the public health policy prioritizing First Nations for vaccination was associated with faster access to the vaccine. See **S4 Table** for more details on the age group data points.

## Discussion

Our study found that testing rates for First Nations were double the rates for AOM during the first 2 waves of the COVID-19 pandemic in Manitoba, and infection rates were 3 to 4 times higher than for AOM. First Nations had higher relative rates of vaccination compared to AOM while they were being prioritized for vaccination. Nearly all First Nations age groups received both their first and second vaccine doses sooner than AOM (an average of 16 and 14 days sooner, respectively). Given that First Nations-led advocacy helped to ensure priority access to vaccinations and culturally safe COVID-19 protective measures, our results suggest that the partnered COVID-19 response between First Nations and the Manitoba government contributed to vaccine acceptance and quick uptake among First Nations.

Although there has been much literature published on COVID-19 in the past 3 years, only a few studies examine the impact of the pandemic on Indigenous peoples in Canada and internationally [3,38–41], and even fewer have attempted to recognize the heterogeneity of Indigenous peoples across Canada using a nations-based approach [42]. Relevant to our study, a rapid critical research review examined vaccination, infection, and mortality rates up to August 2021, and found that Indigenous peoples in Canada and the United States were more likely than non-Indigenous people to be infected, experience severe COVID-19 illness, or to die as a result of their illness; however, the distribution of vaccinations was roughly the same between Indigenous and non-Indigenous populations [3]. Another study looked at infection and vaccination rates among Indigenous (First Nations, Métis, and Inuit) people in 2 cities in Ontario, Canada, finding that (as a group) their infection rates were similar and their vaccination rates were lower than the city and provincial overall rates [43]. The lack of Indigenous identifier collection has been recognized as a major barrier to examining the effects of COVID-19 on Indigenous peoples (and on specific nations) in Canada and worldwide, hindering timely and complete Indigenous-led public health efforts [3,38–40]. In Manitoba, First Nations and government organizations working in public health have been lauded as leaders in this area and have provided an example of a successful collaboration to establish data sovereignty agreements and provide accurate data to inform the development and implementation of evidence-based strategies to combat the spread and severe health outcomes of COVID-19 [44]. Making Indigenous identifier data available has helped provide a clearer picture of the benefits and outcomes of First Nations’ self-determination and public health sovereignty approaches.

Major strengths of our study include a robust analytic design and the use of the comprehensive, population-based data in Manitoba Population Research Data Repository, which allowed us to adjust for potential differences between study groups and made it possible to examine COVID-19 outcomes for First Nations using a nations-based approach. This approach was designed jointly by the First Nations and University of Manitoba researchers leading the study with respect for Indigenous data sovereignty principles [32] and the First Nations principles of Ownership, Control, Access, and Possession (OCAP) [33].

The study also has some notable limitations, such as the possibility that unmeasured confounders influenced the results. We did not have any data available on potential barriers to COVID-19 testing or vaccination, including, for example, social and structural barriers faced by Indigenous and other racialized people when culturally responsive supports are not available [45,46], and the distrust Indigenous people may have in governments due to historical harms, such as medical experimentation in Indigenous communities [45–48]. However, collaborative efforts to develop and implement new testing and vaccine administration strategies to improve access were ongoing throughout the pandemic.

Globally, Indigenous people have had better COVID-19 outcomes in regions where there is not only Indigenous-specific data available, but also where they have been involved in the development and implementation of response plans [40]. In Manitoba, each First Nation organized testing and vaccination clinics in their own community to supplement the provincial public health programming. Some communities delivered the vaccinations with their own complement of nursing staff while others secured additional resources and support through the Rapid Response Teams. Community-specific data were collected by the province but were reported on an online dashboard managed by the Tribal Council Areas (groupings of First Nations), and biweekly updates on social media were provided through collaboration between FNHSSM, the Assembly of Manitoba Chiefs, and Ongomiizwin Health Services. This type of informative, inclusive, and participatory decision-making and policy development is key to preventing the spread of communicable illnesses (including COVID-19) in Indigenous populations [40,44,45]. The structural inequities and systemic racism experienced by Indigenous peoples nationwide have been brought to the forefront by the COVID-19 pandemic [42,45]. Although the results of our analysis provide valuable information to look back on and apply learnings for the future, retrospective studies do not typically provide the real-time information that is often critical in a public health emergency. Even when we look to lessons from the past, such as those from the H1N1 pandemic [4], strong advocacy and collaborative efforts are still needed to ensure equitable access to protective measures for vulnerable populations. Studies like this one are important to ensure First Nations’ voices are heard and to provide important learnings about how Indigenous-led initiatives and partnership-based public health efforts can influence health outcomes.

This study demonstrates how First Nations’ sovereignty over their data and their health priorities can support First Nations health and well-being during a public health emergency. Policies, programs, and public health messaging that prioritize First Nations and provide culturally relevant care can influence the speed of vaccine uptake and help provide important protections against the spread of a virus. To smooth the way for future partnerships and Indigenous self-determination, Canadian governments and health systems need to continue decolonizing health research and healthcare and redress the current and historical colonial structures that negatively impact the health and well-being of Indigenous peoples.

## Supporting information

S1 Fig Test positivity rates for COVID-19 diagnostic testing among First Nations and All Other Manitobans. Monthly moving average rates per 1,000 person-months and 95% CIs. All ages. February 2020–March 2021.
(DOCX)

S1 Table Variable definitions. (DOCX)

S2 Table **Table A.** Crude COVID-19 testing rates among First Nations and All Other Manitobans. Monthly moving averages per 1,000 person-months and 95% CIs, all ages. **Table B.** Crude COVID-19 infection rates among First Nations and All Other Manitobans. Monthly moving averages per 1,000 person-months and 95% CIs, all ages. **Table C.** Crude COVID-19 vaccination rates among First Nations and All Other Manitobans. Monthly moving averages per 1,000 person-months and 95% CIs, all ages.
(DOCX)

S3 Table Crude COVID-19 vaccination rate ratios with 95% CIs. First Nations and All Other Manitobans.
(DOCX)

S4 Table Restricted means survival analysis data table. (DOCX)

## Abbreviations

AOM: All Other Manitobans
COVID-19: Coronavirus Disease 2019
FNHSSM: First Nations Health and Social Secretariat of Manitoba
ICU: intensive care unit
OCAP: Ownership, Control, Access, and Possession
RMST: restricted mean survival time
