# Supplementary material for: COVID-19 diagnostic testing and vaccinations among First Nations in Manitoba: A nations-based retrospective cohort study using linked administrative data, 2020–2021
Source: PLoS Med. 2024 Feb 16;21(2):e1004348. doi: 10.1371/journal.pmed.1004348 (PMC10871479; doi:10.1371/journal.pmed.1004348)
Supplement: S1 Fig — Monthly moving average rates per 1,000 person-months and 95% CIs. All ages. February 2020–March 2021. (DOCX) [file pmed.1004348.s001.docx]

|  | **2020** | | | | | | | | | | | **2021** | | |
| --- | --- | --- | --- | --- | --- | --- | --- | --- | --- | --- | --- | --- | --- | --- |
|  | **Feb** | **Mar** | **Apr** | **May** | **Jun** | **Jul** | **Aug** | **Sep** | **Oct** | **Nov** | **Dec** | **Jan** | **Feb** | **Mar** |
| **First Nations** |  | | | | | | | | | | | | | |
| Test Positivity Rate | 0.15 | 0.27 | 0.27 | 0.12 | 0.01 | 0.04 | 0.54 | 2.70 | 7.79 | 14.09 | 18.29 | 16.98 | 13.28 | 10.37 |
| 95% CI (lower limit) | 0.00 | 0.12 | 0.12 | 0.00 | 0.00 | 0.00 | 0.36 | 2.46 | 7.54 | 13.91 | 18.23 | 16.86 | 13.14 | 10.31 |
| 95% CI (upper limit) | 0.44 | 0.42 | 0.42 | 0.27 | 0.05 | 0.08 | 0.72 | 2.94 | 8.05 | 14.28 | 18.35 | 17.09 | 13.43 | 10.43 |
| **All Other Manitobans** |  | | | | | | | | | | | | | |
| Test Positivity Rate | 0.54 | 0.91 | 0.96 | 0.48 | 0.22 | 0.82 | 1.35 | 2.98 | 6.06 | 8.84 | 9.22 | 6.57 | 4.09 | 2.96 |
| 95% CI (lower limit) | 0.39 | 0.82 | 0.88 | 0.41 | 0.21 | 0.76 | 1.31 | 2.92 | 5.98 | 8.80 | 9.18 | 6.49 | 4.04 | 2.94 |
| 95% CI (upper limit) | 0.69 | 1.01 | 1.04 | 0.56 | 0.24 | 0.88 | 1.39 | 3.03 | 6.14 | 8.89 | 9.26 | 6.64 | 4.15 | 2.97 |

**S1 Fig.** **Test Positivity Rates for COVID-19 Diagnostic Testing among First Nations and All Other Manitobans.**

Monthly moving average rates per 1,000 person-months and 95% Confidence Intervals. All ages. Feb 2020 – Mar 2021.
