# Supplementary material for: COVID-19 diagnostic testing and vaccinations among First Nations in Manitoba: A nations-based retrospective cohort study using linked administrative data, 2020–2021
Source: PLoS Med. 2024 Feb 16;21(2):e1004348. doi: 10.1371/journal.pmed.1004348 (PMC10871479; doi:10.1371/journal.pmed.1004348)
Supplement: S1 Table — (DOCX) [file pmed.1004348.s002.docx]

| **S1 Table. Variable Definitions** | | |
| --- | --- | --- |
| **Variable** | **Data Source(s)** | **Description** |
| **Cohort Identification** | | |
| First Nations identity | Manitoba First Nations Research File, Universal Newborn Screen, Healthy Baby Program Data, Employment and Income Assistance, Early Development Instrument Data | Used to distinguish all people who identify as First Nations or as a mother of a First Nations person versus all other Manitobans |
| **Covariates** | | |
| Date of birth | Manitoba Health Insurance Registry^a^ | Used to determine age/age groups |
| Biological sex | Manitoba Health Insurance Registry | Biological sex assigned at birth, i.e., female or male |
| Postal code | Statistics Canada | Used to determine urban versus rural geography (i.e., region of residence) |
| Average small area-level income | Canada Census | Used to create population-based income quintiles |
| Mental health | Medical Claims, Hospital Discharge Abstract Database,  Drug Program Information Network | Reason for physician visit, hospitalization, or ICU admission (ICD-9 and ICD-10 codes), as well as ATC codes for prescriptions drugs |
| Physical health | Medical Claims, Hospital Discharge Abstract Database,  Drug Program Information Network | Reason for physician visit, hospitalization, or ICU admission (ICD-9 and ICD-10 codes), as well as ATC codes for prescription drugs, used to calculate Charlson Comorbidity Index^b^ |
| **Outcome Measures** | | |
| COVID-19 diagnostic PCR test | COVID-19 Laboratory Test Data from Diagnostic Services Manitoba | Indication for COVID-19 test and diagnostic test results |
| COVID-19 infection | COVID-19 Laboratory Test Data from Diagnostic Services Manitoba | COVID-19 diagnostic test result |
| Vaccination | COVID-19 Surveillance Data from the Public Health Information Management System | COVID-19 vaccination records (vaccine type, number of doses) |
| ^a^ The Manitoba Population Research Data Repository contains administrative records on >99.9% of the Manitoba population. Health records in a few select datasets may be incomplete because they are under federal jurisdiction (e.g., for military personnel, individuals incarcerated in federal prisons and individuals living in First Nations communities).  ^b^ The Charlson Comorbidity Index is a way of categorizing physical comorbidities based on the ICD diagnosis codes. Each comorbidity category has an associated weight (from 1 to 6), based on the adjusted risk of mortality or resource use, and the sum of all the weights results in a single comorbidity score for a patient. A score of zero indicates that no comorbidities were found. The higher the score, the more likely the predicted outcome will result in mortality or higher resource use. | | |
|  | | |
