# Supplementary material for: COVID-19 diagnostic testing and vaccinations among First Nations in Manitoba: A nations-based retrospective cohort study using linked administrative data, 2020–2021
Source: PLoS Med. 2024 Feb 16;21(2):e1004348. doi: 10.1371/journal.pmed.1004348 (PMC10871479; doi:10.1371/journal.pmed.1004348)
Supplement: S2 Table — Table A. Crude COVID-19 testing rates among First Nations and All Other Manitobans. Monthly moving averages per 1,000 person-months and 95% CIs, all ages. Table B. Crude COVID-19 infection rates among First Nations and All Other Manitobans. Monthly moving averages per 1,000 person-months and 95% CIs, all ages. Table C. Crude COVID-19 vaccination rates among First Nations and All Other Manitobans. Monthly moving averages per 1,000 person-months and 95% CIs, all ages. (DOCX) [file pmed.1004348.s003.docx]

| **Table S2-a. Crude COVID-19 Testing Rates Among First Nations And All Other Manitobans.** Monthly Moving Averages per 1,000 Person-Months and 95% Confidence Intervals, All Ages. | | | | | | | | |
| --- | --- | --- | --- | --- | --- | --- | --- | --- |
|  | **First Nations**  **N=144,816** | | | | **All Other Manitobans**  **N=1,262,760** | | | |
| **Month-Year** | **No. of Tests** | **Testing Rate** | **95% CI**  **(lower limit)** | **95% CI**  **(upper limit)** | **No. of Tests** | **Testing Rate** | **95% CI**  **(lower limit)** | **95% CI**  **(upper limit)** |
| **Jan-20** | 0 | 0.00 | 0.00 | 0.01 | . | 0.03 | 0.02 | 0.03 |
| **Feb-20** | . | 1.54 | 1.34 | 1.74 | 62 | 2.50 | 2.41 | 2.58 |
| **Mar-20** | 668 | 6.05 | 5.79 | 6.31 | 9238 | 6.09 | 6.02 | 6.16 |
| **Apr-20** | 1968 | 11.96 | 11.78 | 12.14 | 13401 | 10.48 | 10.45 | 10.51 |
| **May-20** | 2573 | 17.14 | 17.06 | 17.22 | 16460 | 12.27 | 12.26 | 12.28 |
| **Jun-20** | 2913 | 19.97 | 19.92 | 20.01 | 15892 | 15.06 | 15.03 | 15.09 |
| **Jul-20** | 3182 | 24.58 | 24.46 | 24.69 | 23757 | 21.62 | 21.55 | 21.69 |
| **Aug-20** | 4561 | 29.63 | 29.51 | 29.75 | 40910 | 28.22 | 28.17 | 28.27 |
| **Sep-20** | 5102 | 46.72 | 46.41 | 47.04 | 40598 | 38.67 | 38.61 | 38.72 |
| **Oct-20** | 10579 | 68.44 | 68.09 | 68.80 | 62858 | 49.15 | 49.08 | 49.23 |
| **Nov-20** | 13963 | 87.63 | 87.50 | 87.76 | 80212 | 51.49 | 51.43 | 51.54 |
| **Dec-20** | 13357 | 94.20 | 94.18 | 94.22 | 49364 | 45.09 | 45.01 | 45.17 |
| **Jan-21** | 13378 | 85.97 | 85.85 | 86.10 | 38966 | 32.89 | 32.85 | 32.92 |
| **Feb-21** | 10360 | 81.93 | 81.82 | 82.04 | 34662 | 31.18 | 31.16 | 31.19 |
| **Mar-21** | 11609 | 83.83 | 83.69 | 83.97 | 43116 | 37.28 | 37.22 | 37.34 |
| **Apr-21** | 14199 | 99.32 | 99.15 | 99.50 | 62110 | 50.67 | 50.59 | 50.74 |
| **May-21** | 17040 | 103.19 | 103.06 | 103.32 | 85273 | 50.77 | 50.70 | 50.85 |
| **Jun-21** | 13249 | 89.72 | 89.42 | 90.02 | 43760 | 43.01 | 42.91 | 43.12 |
| **Jul-21** | 8375 | 68.01 | 67.77 | 68.25 | 33127 | 31.10 | 31.07 | 31.12 |
| **Aug-21** | 7676 | 60.35 | 60.25 | 60.45 | 40636 | 36.33 | 36.26 | 36.39 |
| **Sep-21** | 9974 | 71.40 | 71.19 | 71.61 | 63988 | 45.58 | 45.52 | 45.63 |
| **Oct-21** | 13146 | 83.59 | 83.47 | 83.72 | 68540 | 52.84 | 52.83 | 52.85 |
| **Nov-21** | 13885 | 95.88 | 95.79 | 95.96 | 74541 | 59.03 | 58.99 | 59.07 |
| **Dec-21** | 142050 | 98.09 | 97.96 | 98.22 | 77685 | 61.52 | 61.47 | 61.57 |

| **Table S2-b. Crude COVID-19 Infection Rates Among First Nations and All Other Manitobans.** Monthly Moving Averages per 1,000 Person-Months and 95% Confidence Intervals, All Ages. | | | | | | | | |
| --- | --- | --- | --- | --- | --- | --- | --- | --- |
|  | **First Nations**  **N=144,816** | | | | **All Other Manitobans**  **N=1,262,760** | | | |
| **Month-Year** | **No. of Positive Tests** | **Infection Rate** | **95% CI**  **(lower limit)** | **95% CI**  **(upper limit)** | **No. of Positive Tests** | **Infection Rate** | **95% CI**  **(lower limit)** | **95% CI**  **(upper limit)** |
| **Jan-20** | 0 | 0.00 | 0.00 | 0.00 | . | . | . | . |
| **Feb-20** | . | 0.01 | 0.00 | 0.02 | 0 | 0.04 | 0.03 | 0.05 |
| **Mar-20** | . | 0.02 | 0.01 | 0.04 | 149 | 0.08 | 0.07 | 0.09 |
| **Apr-20** | 7 | 0.02 | 0.01 | 0.04 | 152 | 0.09 | 0.08 | 0.09 |
| **May-20** | 0 | 0.02 | 0.00 | 0.04 | 22 | 0.05 | 0.05 | 0.06 |
| **Jun-20** | 0 | 0.00 | 0.00 | 0.01 | 29 | 0.04 | 0.03 | 0.04 |
| **Jul-20** | . | 0.01 | 0.00 | 0.02 | 85 | 0.24 | 0.22 | 0.26 |
| **Aug-20** | . | 0.19 | 0.12 | 0.25 | 787 | 0.43 | 0.41 | 0.44 |
| **Sep-20** | 76 | 1.77 | 1.59 | 1.96 | 718 | 1.28 | 1.25 | 1.32 |
| **Oct-20** | 688 | 6.72 | 6.45 | 6.99 | 3293 | 3.47 | 3.41 | 3.53 |
| **Nov-20** | 2145 | 12.86 | 12.67 | 13.05 | 8962 | 4.62 | 4.58 | 4.65 |
| **Dec-20** | 2725 | 17.20 | 17.15 | 17.24 | 4994 | 4.40 | 4.36 | 4.44 |
| **Jan-21** | 2554 | 14.98 | 14.84 | 15.12 | 2477 | 2.30 | 2.26 | 2.33 |
| **Feb-21** | 1180 | 11.17 | 11.01 | 11.33 | 1116 | 1.27 | 1.26 | 1.29 |
| **Mar-21** | 1078 | 8.14 | 8.12 | 8.16 | 1165 | 1.52 | 1.49 | 1.54 |
| **Apr-21** | 1251 | 10.75 | 10.62 | 10.89 | 3413 | 3.75 | 3.70 | 3.81 |
| **May-21** | 2304 | 12.29 | 12.19 | 12.39 | 9550 | 4.33 | 4.29 | 4.37 |
| **Jun-21** | 1735 | 10.79 | 10.62 | 10.96 | 3344 | 3.67 | 3.61 | 3.72 |
| **Jul-21** | 607 | 6.11 | 5.90 | 6.31 | 921 | 1.35 | 1.32 | 1.38 |
| **Aug-21** | 288 | 2.90 | 2.84 | 2.97 | 830 | 0.89 | 0.88 | 0.90 |
| **Sep-21** | 358 | 3.69 | 3.57 | 3.81 | 1613 | 1.21 | 1.19 | 1.22 |
| **Oct-21** | 948 | 5.14 | 5.04 | 5.23 | 2142 | 1.92 | 1.90 | 1.94 |
| **Nov-21** | 1116 | 8.40 | 8.29 | 8.51 | 9560 | 4.72 | 4.66 | 4.79 |
| **Dec-21** | 1348 | 9.31 | 9.15 | 9.47 | 12530 | 6.24 | 6.16 | 6.32 |

| **Table S2-c. Crude COVID-19 Vaccination Rates Among First Nations and All Other Manitobans.** Monthly Moving Averages per 1,000 Person-Months and 95% Confidence Intervals, All Ages. | | | | | | | | |
| --- | --- | --- | --- | --- | --- | --- | --- | --- |
|  | **First Nations**  **N=144,816** | | | | **All Other Manitobans**  **N=1,262,760** | | | |
| **Month-Year** | **No. of Vaccinations** | **Vaccination Rate** | **95% CI**  **(lower limit)** | **95% CI**  **(upper limit)** | **No. of Vaccinations** | **Vaccination Rate** | **95% CI**  **(lower limit)** | **95% CI**  **(upper limit)** |
| **Dec-20** | 16 | 19.10 | 18.40 | 19.80 | 774 | 15.01 | 14.84 | 15.19 |
| **Jan-21** | 4170 | 21.43 | 21.04 | 21.82 | 13430 | 18.58 | 18.48 | 18.68 |
| **Feb-21** | 2812 | 55.63 | 55.11 | 56.16 | 17182 | 50.83 | 50.64 | 51.02 |
| **Mar-21** | 13404 | 105.49 | 104.75 | 106.23 | 104668 | 107.52 | 107.25 | 107.79 |
| **Apr-21** | 26466 | 170.27 | 169.83 | 170.70 | 237919 | 188.91 | 188.71 | 189.11 |
| **May-21** | 31308 | 216.62 | 216.44 | 216.80 | 334761 | 291.05 | 290.85 | 291.25 |
| **Jun-21** | 34395 | 210.52 | 210.28 | 210.77 | 499241 | 312.12 | 311.97 | 312.27 |
| **Jul-21** | 24152 | 167.09 | 166.53 | 167.64 | 326677 | 240.87 | 240.51 | 241.23 |
| **Aug-21** | 12871 | 113.30 | 112.87 | 113.73 | 70420 | 120.68 | 120.33 | 121.04 |
| **Sep-21** | 11453 | 82.16 | 82.08 | 82.23 | 52810 | 51.81 | 51.78 | 51.83 |
| **Oct-21** | 8458 | 85.07 | 84.94 | 85.19 | 37168 | 52.19 | 52.16 | 52.21 |
| **Nov-21** | 9934 | 106.90 | 106.57 | 107.23 | 74856 | 114.72 | 114.41 | 115.02 |
| **Dec-21** | 7485 | 122.52 | 122.17 | 122.87 | 68264 | 145.09 | 144.69 | 145.50 |
