# Supplementary material for: COVID-19 diagnostic testing and vaccinations among First Nations in Manitoba: A nations-based retrospective cohort study using linked administrative data, 2020–2021
Source: PLoS Med. 2024 Feb 16;21(2):e1004348. doi: 10.1371/journal.pmed.1004348 (PMC10871479; doi:10.1371/journal.pmed.1004348)
Supplement: S3 Table — First Nations and All Other Manitobans. (DOCX) [file pmed.1004348.s004.docx]

| **Table S3. Crude COVID-19 Vaccination Rate Ratios with 95% Confidence Intervals.** First Nations and All Other Manitobans. | | | | |
| --- | --- | --- | --- | --- |
| **Month** | **Crude**  **Rate Ratio** | **95% CI** | **Adjusted Rate Ratio** | **95% CI** |
| December 2020 | 0.14 | 0.01-2.31 | 0.24 | 0.18-0.33 |
| January 2021 | 1.39 | 0.09-22.15 | 1.80 | 1.61-2.00 |
| February 2021 | 1.01 | 0.06-16.22 | 1.29 | 1.15-1.44 |
| March 2021 | 1.04 | 0.06-16.56 | 1.14 | 1.03-1.25 |
| April 2021 | 0.95 | 0.06-15.18 | 1.15 | 1.04-1.26 |
| May 2021 | 0.82 | 0.05-13.06 | 1.06 | 0.97-1.17 |
| June 2021 | 0.60 | 0.04-9.54 | 0.71 | 0.65-0.78 |
| July 2021 | 0.65 | 0.04-10.37 | 0.76 | 0.69-0.83 |
| August 2021 | 1.61 | 0.10-25.67 | 1.63 | 1.47-1.80 |
| September 2021 | 1.78 | 0.11-28.47 | 1.76 | 1.59-1.95 |
| October 2021 | 1.40 | 0.09-22.41 | 1.53 | 1.38-1.69 |
| November 2021 | 1.73 | 0.11-27.60 | 1.13 | 1.02-1.25 |
| December 2021 | 0.63 | 0.04-10.03 | 0.79 | 0.72-0.87 |
| Adjusted rate ratios were adjusted for age, sex, income, region of residence, mental health conditions, and Charlson Comorbidity Index. | | | | |
