# Supplementary material for: COVID-19 diagnostic testing and vaccinations among First Nations in Manitoba: A nations-based retrospective cohort study using linked administrative data, 2020–2021
Source: PLoS Med. 2024 Feb 16;21(2):e1004348. doi: 10.1371/journal.pmed.1004348 (PMC10871479; doi:10.1371/journal.pmed.1004348)
Supplement: S4 Table — (DOCX) [file pmed.1004348.s005.docx]

| **Table S4. Restricted Means Survival Analysis Data Table** | | | | | | |  |
| --- | --- | --- | --- | --- | --- | --- | --- |
|  | **First Nations** | | | **All Other Manitobans** | | |  |
|  | **Vaccinations** | **Population** | **Time Estimate**  **(Days, 95% CI)** | **Vaccinations** | **Population** | **Time Estimate**  **(Days, 95% CI)** | **Difference in**  **Time Estimate**  **(Days, 95% CI)** |
| **First Vaccination** | | | | | | |  |
| Overall | 107,971 | 143,822 | 184.96  (184.36, 185.57) | 970,050 | 1,245,876 | 200.44  (200.02, 200.82) | -15.47 (-16.00, -14.94) |
| Age 0-9 | 11,614 | 31,124 | 352.58  (351.98, 353.19) | 57,796 | 160,311 | 363.22  (362.79, 363.64) | -10.63 (-11.15, -10.12) |
| Age 10-19 | 24,725 | 29,062 | 216.84  (215.78, 217.90) | 114,286 | 140,695 | 227.83  (227.27, 228.39) | -10.99 (-12.09, -9.89) |
| Age 20-29 | 21,108 | 25,777 | 206.66  (205.36, 207.97) | 127,146 | 163,598 | 221.19  (220.59, 221.78) | -14.52 (-15.87, -13.18) |
| Age 30-39 | 15,561 | 18,620 | 197.51  (196.00, 199.03) | 141,094 | 175,041 | 210.60  (210.03, 211.17) | -13.08 (-14.63, -11.54) |
| Age 40-49 | 13,056 | 14,910 | 175.99  (174.37, 177.60) | 132,798 | 157,677 | 195.48  (194.91, 196.06) | -19.50 (-21.16, -17.84) |
| Age 50-59 | 11,877 | 13,180 | 157.86  (156.2, 159.52) | 140,326 | 161,600 | 180.04  (179.50, 180.59) | -22.18 (-23.89, -20.48) |
| Age 60-69 | 6,727 | 7,393 | 132.31  (130.04, 134.58) | 130,392 | 146,164 | 164.57  (164.05, 165.09) | -32.26 (-34.56, -29.97) |
| Age 70-79 | 2,617 | 2,918 | 120.97  (117.12, 124.81) | 81,903 | 90,273 | 152.78  (152.19, 153.37) | -31.81 (-35.68, -27.94) |
| Age 80-89 | 633 | 758 | 128.59  (120.12, 137.07) | 35,601 | 39,937 | 143.54  (142.66, 144.43) | -14.95 (-23.47, -6.43) |
| Age 90+ | 53 | 80 | 174.47  (145.63, 203.32) | 8,708 | 10,580 | 146.33  (144.27, 148.38) | 28.15 (-0.77, 57.06) |
|  | | | | | | |  |
| **Second Vaccination** | | | | | | |  |
| Overall | 68,342 | 10,7971 | 319.75  (319.02, 334.11) | 414,726 | 970,050 | 333.63  (333.15, 334.11) | -13.88 (-14.50, -13.26) |
| Age 0-9 | 3,499 | 11,614 | 411.45  (410.40, 412.50) | 14,245 | 57,796 | 410.64  (410.08, 411.20) | 0.81 (-0.22, 1.84) |
| Age 10-19 | 21,226 | 24,725 | 273.02  (271.91, | 94,309 | 114,286 | 266.89  (266.28, 267.50) | 6.13 (5.01, 7.25) |
| Age 20-29 | 15,852 | 21,108 | 280.88  (279.50, 274.12) | 77,273 | 127,146 | 298.15  (297.49, 298.80) | -17.27 (-18.67, -15.87) |
| Age 30-39 | 10,681 | 15,561 | 288.85  (287.27, 282.25) | 76,099 | 141,094 | 307.04  (306.41, 307.67) | -18.20 (-19.79, -16.60) |
| Age 40-49 | 7,752 | 13,056 | 296.66  (294.90, 290.43) | 59,917 | 132,798 | 317.82  (317.19, 318.44) | -21.16 (-22.94, -19.38) |
| Age 50-59 | 5,764 | 11,877 | 309.31  (307.46, 298.41) | 44,707 | 140,326 | 337.27  (336.69, 337.85) | -27.96 (-29.83, -26.08) |
| Age 60-69 | 2,452 | 6,727 | 323.96  (321.45, 311.17) | 26,868 | 130,392 | 353.76  (353.22, 354.31) | -29.80 (-32.32, -27.28) |
| Age 70-79 | 863 | 2,617 | 323.96  (319.7, 326.47) | 13,159 | 81,903 | 359.87  (359.28, 360.46) | -35.90 (-40.18, -31.62) |
| Age 80-89 | 235 | 633 | 304.29  (294.50, 328.23) | 6,117 | 35,601 | 354.38  (353.49, 355.27) | -50.09 (-59.90, -40.27) |
| Age 90+ | 18 | 53 | 311.81  (278.93, 314.08) | 2,032 | 8,708 | 331.07  (328.79, 333.36) | -19.26 (-52.22, 13.70) |
